# The antioxidant effect of resveratrol on leukocytes from patients with Alzheimer is independent of SIRT1 signaling pathway

**DOI:** 10.1016/j.bbrep.2025.102291

**Published:** 2025-10-01

**Authors:** Filipe Nogueira Franco, Luciana de Cassia Cardoso, Bárbara Néllita Moura Silva, Glaucy Rodrigues de Araújo, Miriam Martins Chaves

**Affiliations:** Biochemistry Laboratory of Aging and Correlated Diseases, Department of Biochemistry and Immunology, Biological Sciences Institute, Federal University of Minas Gerais, Av. Antônio Carlos 6627, CP 486, 30161-970, Belo Horizonte, MG, Brazil

**Keywords:** Alzheimer, Functionality, Oxidative stress, Resveratrol, SIRT1

## Abstract

Alzheimer's Disease (AD) is the most prevalent dementia in aging. Among its aspects is cognitive and functional decline, resulting from an increase in Reactive Oxygen Species (ROS) and Nitrogen (RNS). Resveratrol (RSV) is a polyphenolic compound that has been recognized as a potent antioxidant. The objective was to verify the oxidative profile of AD in leukocytes, correlating the main oxidative parameters with the functionality of these elderly individuals and verify the antioxidant effect of RSV. For this, ROS and RNS, the antioxidant enzymes catalase and glutathione peroxidase (GPx), as well as the action of the SIRT1 on leukocytes of elderly people without and with AD, in the presence and absence of RSV, were evaluated. It was observed that RSV, despite acting in the AD group, had its antioxidant power reduced compared to the group without AD. RSV was able to increase GPx in both groups. Analyzing SIRT1, we observe the silencing of this signaling pathway in leukocytes from AD. AD was more dependent on the Katz index. Therefore, we observed that oxidative stress predisposes to an increased loss of autonomy and independence in AD and that the antioxidant effect of RSV is reduced.

## Highlights

• Alzheimer's disease is the most prevalent type of dementia in the elderly.
• The loss of functional capacity may be related to biochemical changes, for example increased oxidative stress.
• Resveratrol is a natural polyphenol that acts as an antioxidant through specific cellular signaling pathways.

## 1 Introduction

Age represents the greatest risk factor for the development of Alzheimer's Disease (AD). As the population continues to age, AD cases will increase and become the most prevalent dementia. As one of the causes of aging, the free radical theory predicts that these are directly related to genetic and behavioural modifications and postulates that free radical reactions are involved in age-related disorders. The accumulation of free radicals, such as hydrogen peroxide (H_2_O_2_), hydroxyl radical (OH•) and reactive nitrogen species (NO and ONOO-), leads to oxidative stress. In addition, copper is a potent mediator of the hydroxyl radical, contributing to the increased oxidative stress characteristic of AD [[1], [2], [3]].

The oxidative stress has been associated with the length of Aβ fragments, with Aβ (1–42) being more toxic than Aβ (1–40) and therefore the most likely candidate for generating hydrogen peroxide and other ROS in this disease [4]. Elevated production of amyloid-beta (A beta) as a preventive antioxidant for brain lipoproteins under the action of increased oxidative stress in aging is postulated to represent a major event in the development of AD. Increase in A beta production is followed by chelation of transition metal ions by A beta, accumulation of A beta-metal lipoprotein aggregates, production of reactive oxygen species and neurotoxicity [5]. In contrast to pro-oxidative properties, antioxidative activity of Aβ peptides has been barely studied. This is somewhat surprising, in view of the fact that strong metal chelators typically are strong antioxidants, and that the metal-binding properties of Aβ have long been known. Exogenously added Aβ has been demonstrated to inhibit metal-catalyzed oxidation of lipoproteins from human cerebrospinal fluid (CSF) and plasma [6]. In contrast, all Aβ peptides are unable to considerably influence metal-independent lipoprotein oxidation, suggesting that the antioxidative activity of Aβ is mainly mediated by chelating transition metal ions by its hydrophilic moiety rather than by free radical scavenging through Met35. Nevertheless, the latter mechanism contributes to the antioxidative activity of Aβ, because Aβ25-35 is able to inhibit lipid peroxidation (at low, compared to lipids, concentrations of the peptide) and since replacement of Met35 by Leu considerably weakens the effect [7].

Although AD is likely associated with multiple etiologies and pathophysiological mechanisms, oxidative stress appears to be an important component of the pathophysiological process [8]. However, the mechanisms underlying oxidative stress, as well as its initiation, remain poorly studied. It is known, for example, that oxidative stress participates in the development of AD by increasing Aβ deposition and by promoting the hyperphosphorylation of tau protein and, consequently, the loss of synapses and neurons, which also induces oxidative stress. This mechanism leads to a vicious cycle [9].

Functional incapacity and cognitive decline may also occur as a worsening of the dementia condition, reflecting on the reduced ability to maintain the physical and mental skills necessary for an independent and autonomous life. According to Souza et al. [3], although there is currently no cure for AD, the adoption of a healthier lifestyle has been associated with a reduction in the decline cognitive impairment, disability and even the risk of dementia (including AD). Strategies include hypocaloric diet, physical exercise and food intake antioxidants, which are associated with neuroprotective mechanisms. Resveratrol is one such antioxidant that has been studied and may contribute to its effects on AD [10].

Some studies have demonstrated numerous biological and pharmacological effects, as cardioprotective, anticancer, antiobesity, neuroprotective, anti-inflammatories, antidiabetics and antioxidants [[11], [12], [13], [14]] As for the antioxidant aspect, RSV has a dual effect: it increases the activity of antioxidant enzymes such as glutathione peroxidase (GPx), glutathione S- transferase (GST) and glutathione reductase (GSR) and can act directly with free radicals, removing them [15].

Another possible mechanism by which RSV mediates neuroprotective effects is through the activation of the sirtuin 1 pathway (SIRT1), which plays an essential role in regulating cell function by deacetylating important substrates in neurodegenerative diseases. SIRT-1 also inhibits the activation of NF-κB signaling pathway and can protect neurons against βA toxicity [3,16]. So, the aim of this study was to analyze oxidative stress in leukocytes from elderly people with and without Alzheimer's disease and its comparison with functionality, and the possible modulator and/or neuroprotective effect of Resveratrol on these markers.

## 2 Methodology

### 2.1 Donor Selection

The project was approved by the Ethics and Research Committee of the Federal University of Minas Gerais - CAAE: 14846619.7.0000.5149. Men over 60 years old were selected and separated into two groups: without (control group) and with Alzheimer (AD). Demographic characteristics of patients without Alzheimer's: Age (years old) 76.3 ± 7; Body mass index (kg/m^2^): 23.4 ± 3; Serum Glucose (mg/dL): 102.6 ± 11.3; Serum Triglycerides (mg/dL): 164.3 ± 20 and Serum creatine (mg/dL): 0.741 ± 0.17. As for patients with Alzheimer's, it is: Age (years old) 78 ± 5.4; Body mass index (kg/m^2^): 25.5 ± 4.0; Serum Glucose (mg/dL): 105.6 ± 10.6; Serum Triglycerides (mg/dL): 153.5 ± 23 and Serum creatine (mg/dL): 0.994 ± 0.169. There was no statistical difference in the evaluated parameters comparing the two groups. Furthermore, none of them had cardiovascular complications [17]. Patients (and in some cases their guardians) eligible to participate in the study were invited to sign the Informed Consent Form. A copy of the detailed medical history and other biochemical tests were made available. In total, 10 patients were selected for the AD group, according to Dr. Rafael Pacheco Terra (CRM: 45302) establishing some criteria such as: physiological evaluation; cognitive functions; and mental health (Mini Mental). It is worth noting that the criteria used were the same as those of the National Institute of Neurological and Communicative Disorders and Stroke – Alzheimer's Disease and related Disorders Association (NINCDS-ADRDA), and these patients did not present any other important pathology [4].

### 2.2 Obtaining leukocytes and treatments

Leukocytes were isolated according to the technique described in the literature, with adaptativos [18]. The blood was centrifuged with Leucopaque® and leukocytes were separated by density difference. The cell concentration was 1 × 10^7^ cells/mL in PBS saline solution. The experimental groups were: Control, Resveratrol, H_2_O_2_ and H_2_O_2_+Resveratrol (C, RSV, C+ and RSV+, respectively). The concentrations of H_2_O_2_ (150 μM) and RSV (5 μM) used in the *in vitro* assays were defined by our research group in previous study [19].

### 2.3 Antioxidant enzyme dosage - catalase and glutathione peroxidase (GPx)

According to the experimental group, 1x106 cells were obtained by centrifugation and added to tubes with the respective treatments described above. The cells remained in a water bath at 37 °C for 1 h. After centrifugation, the supernatant was discarded and the cells were resuspended in PBS. After a new centrifugation, the cells were resuspended in ice-cold lysis buffer from the kits. The cells remained on ice for 10 min. After a new centrifugation, the supernatant was collected for the measurement of two of the three main enzymes of the antioxidant complex: catalase (EnzyChromTM catalase assay kit (ECAT-100) - Bioassay systems) and GPx activity (Cayman chemical glutathione peroxidase assay kit No. 703102). The detailed methodology was performed according to the manufacturers' protocol. It is worth mentioning that the superoxide dismutase (SOD) measurement has already been published in the previous study [17].

### 2.4 ROS evaluation - chemiluminescence assay

The quantitative ROS determination was performed through the luminol-dependent chemiluminescence assay, according to HORTA et al. [21] with some adaptations. All tubes with respective treatment were placed in a Luminometer (Lumat, LB 9501, EG&G Berthold, Germany) and readed for 10 min. The results were expressed in relative light units (RLU)/minute.

### 2.5 The SIRT1 signaling pathway analysis

The SIRT1 signaling pathway was analyzed using the luminol-dependent chemiluminescence assay [21]. Sirtinol (10 μM) was used as the specific SIRT1 inhibitor [20]. Briefly, 1 × 10^6^ leukocytes were previously incubated for 30 min in the absence (control group) and presence of inhibitor. After this time, the tubes were centrifuged at 600g for 10 min. The cells were resuspended in PBS and the same treatments with RSV and procedures described above were added.

### 2.6 Nitric Oxide (NO) and peroxynitrite (ONOO^−^) evaluation

After the treatments described above, the cells were centrifuged. The supernatant was removed and stored for NO analysis and the pellet was resuspended in MilliQ H_2_O water for ONOO- quantification. NO quantification was assessed indirectly through nitrite production, largely performed by the GRIESS reaction [22]. Peroxynitrite quantification was performed using the HUGHES & NICKLIN technique [23], by analyzing the absorbance of the samples at a wavelength of 302 nm, capable of detecting the presence of this molecule.

### 2.7 Functionality evaluation through the Katz Index (KI)

The functional capacity of the elderly can be performed by functionality tracking tests, based on basic activities of daily living - ADL, thus assessing their degree of independence and autonomy. The investigated activities are related to bathing, dressing, how to use the bathroom, how to control the sphincter and how to eat, which according to the score, receives a classification according to functionality, through questions asked with the elderly or their caregiver. Its score ranges from 0 to 6, where 6 (maximum score) expresses total independence, and 0 (minimum score) total dependence [24].

### 2.8 Statistical analysis

All results were analyzed using GraphPad Prism software version 9.0 (San Diego, California, USA). First, the Kolmogorov-Smirnov normality test was performed followed by univariate analysis of variance (one-way ANOVA). Dunnett's or Bonferroni's test was performed when the samples showed normal distribution. Dunn's test was performed when they did not follow the normal distribution. p < 0.05 was considered significant. On the other hand, the comparison between functionality and biochemical parameters at baseline levels of the groups without and with Alzheimer's disease was performed using the Mamm-Whitney test.

## 3 Results

### 3.1 Glutathione peroxidase is the main antioxidant enzyme responsible for the effect of RSV in AD

**Fig. 1:**
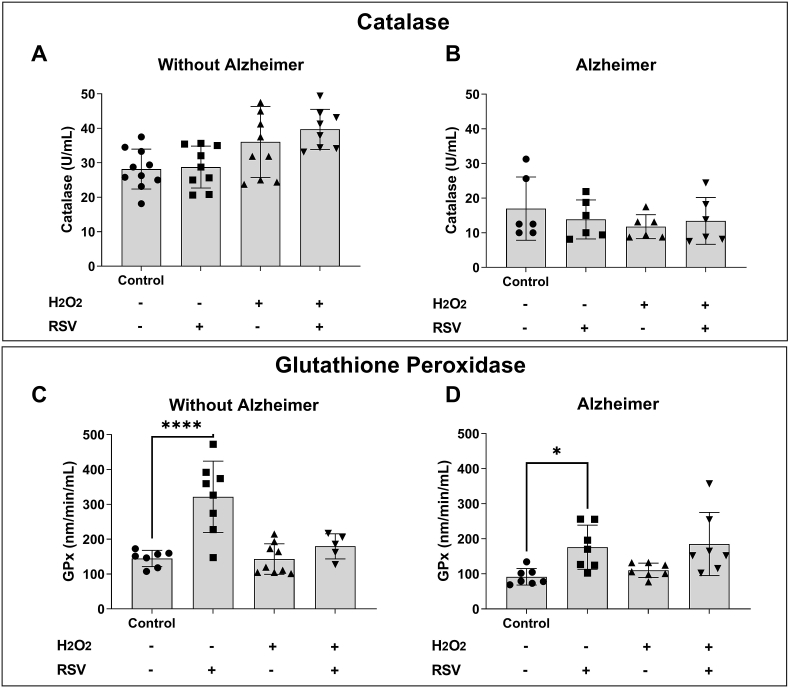
**Evaluation of the effect of resveratrol on the activity of antioxidant enzymes in leukocytes from donors with and without AD.** Panel (A) catalase measurement in leukocytes from elderly individuals without AD and Panel (B) catalase measurement in leukocytes from elderly individuals with AD. Catalase activity was measured in U/mL (Atomic Mass Unit/mL). Panel (C) glutathione peroxidase measurement in leukocytes from elderly individuals without AD and Panel (D) glutathione peroxidase measurement in leukocytes from elderly individuals with AD. Glutathione peroxidase activity was measured in nm/min/mL. An oxidizing environment was induced with the addition of 150 μM hydrogen peroxide (H_2_O_2_); RSV = resveratrol. Statistically significant differences are ∗∗∗∗p < 0.0001 and ∗p < 0.05 through the ANOVA test and Bonferroni Multiple Comparison post-test (n = 10/group).

The first step of our study was to evaluate the effect of two important antioxidant enzymes of elderly people, with and without AD, leukocytes, as well as to evaluate the effect of RSV (Fig. 1). Fig. 1A and B shows that there was no statistical difference between groups, whether exposed to an oxidizing environment (with H_2_O_2_) and/or exposed with H_2_O_2_ and RSV. However, it is possible to observe lower activity for this enzyme in the entire AD group (Fig. 1B).

When we analyze the enzyme Glutathione Peroxidase (GPx), the same profile is observed both in the group without AD and in the AD group. Fig. 1C and D shows that there was an increase in this enzyme activity when leukocytes were treated with RSV. It should be noted that the statistical difference decreases in the AD group, in addition to the fact that RSV cannot increase this enzyme activity in the same way as in the leukocytes of the group without the disease.

### 3.2 RSV has an antioxidant effect in AD independent of SIRT1

After observing the decrease in antioxidant enzymes during AD and the effect of RSV on GPx, ROS production in leukocytes from both groups was evaluated. For this, the SIRT1 pathway inhibitor - sirtinol (10 μM) was added.

**Fig. 2:**
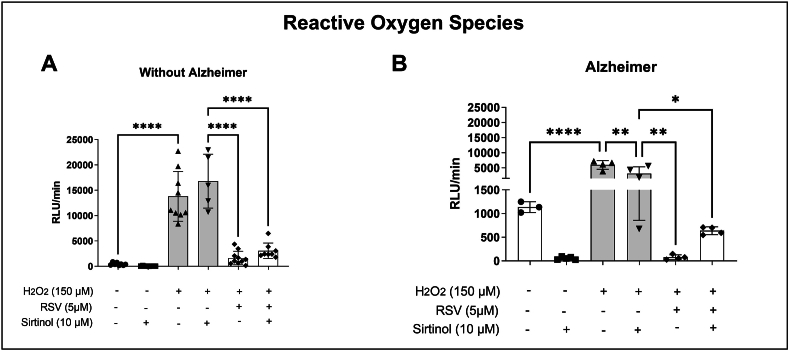
**Evaluation of the antioxidant effect of resveratrol on the SIRT1 cell signaling pathway in leukocytes from elderly individuals with and without AD.** The cells were previously incubated for 30 min with 10 μM sirtinol. After that, they were centrifuged and resuspended in 100 μL of PBS buffer and the treatments were added. Panel (A) shows the value of Relative Light Units (RLU)/minute when the compounds H_2_O_2_ and/or resveratrol (RSV) were added to leukocytes from elderly individuals without AD. Panel (B) shows the RLU/min value when the compounds H_2_O_2_ and/or RSV were added to leukocytes from elderly individuals with AD. The results were significant according to the ANOVA test followed by Tukey's post-test. Statistically significant differences are ∗∗∗∗p < 0.0001; ∗p < 0.05 and ^####^p < 0.0001; ^##^p < 0.001 referring to the H_2_O_2_ group (n = 10/group).

Fig. 2 shows that when leukocytes from the group without AD (Fig. 2A) were exposed to H_2_O_2_, there was a significant increase in ROS production compared to the group without any stimulus. When H_2_O_2_+RSV was added, there was a reduction in ROS compared to leukocytes only stimulated with H_2_O_2_. In leukocytes treated with sirtinol and subsequently exposed to H_2_O_2_+RSV, a significant reduction in ROS production was observed compared to the group treated with sirtinol + H_2_O_2_.

Analyzing the AD group (Fig. 2B) we initially observed a similar pattern in relation to the group without the disease. It was possible to observe a significant increase in ROS when we stimulated leukocytes with H_2_O_2_, when compared with the group without any stimulus. In the H_2_O_2_+RSV group there was a reduction of ROS when compared only to leukocytes stimulated with H_2_O_2_. Still in the AD group, a significant reduction of ROS was observed when the cells were previously treated with sirtinol and subsequently stimulated with H_2_O_2_, when compared with the group that received only H_2_O_2_, indicating that SIRT1 is an active antioxidant pathway. In leukocytes treated with sirtinol and subsequently stimulated with H_2_O_2_+RSV, we observed a reduction in ROS production compared to the sirtinol + H_2_O_2_ group. This is the same pattern observed in leukocytes from donors without AD.

### 3.3 SIRT1 is an important signaling pathway in NO production in AD

**Fig. 3:**
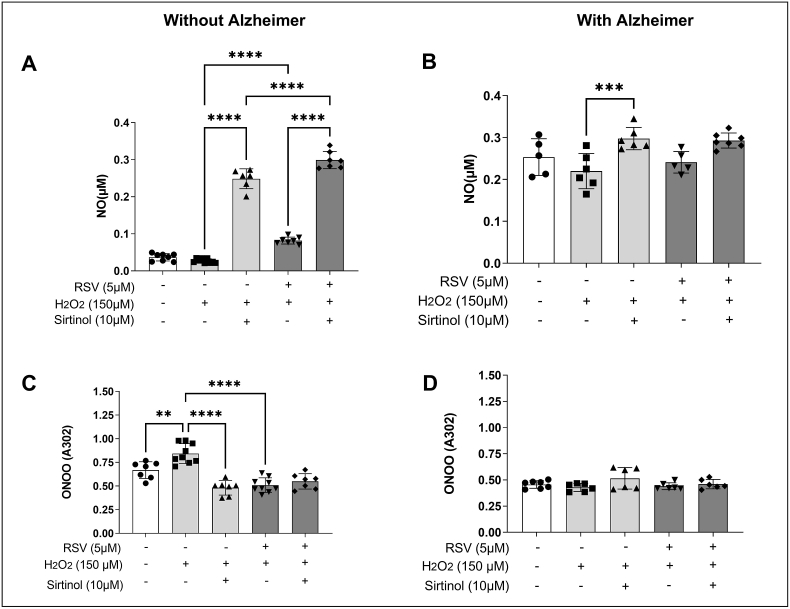
**Evaluation of the effect of resveratrol on the SIRT1 in the production of reactive nitrogen species (RNS) in leukocytes from elderly individuals with and without AD.** The cells were previously incubated for 30 min with 10 μM sirtinol. After that, they were centrifuged and resuspended in 100 μL of PBS buffer, and the treatments were added. Panel (A) Nitric oxide (NO) concentration by the Griess method in leukocytes from patients without AD. Panel (B) NO concentration by the Griess method in leukocytes from patients with AD. The NO concentration was expressed in μM. Panel (C) Peroxynitrite (ONOO^−^) concentration by Hughes & Nicklin method in leukocytes from patients without AD. Panel (D) ONOO^−^ by Hughes & Nicklin method in leukocytes from patients with AD. The ONOO^−^ concentration was expressed as OD at 302 nm. Statistically significant differences are ∗∗p < 0.01; ^&&&&^p < 0.0001; ^##^p < 0.001 and ^####^p < 0.0001 compared to H_2_O_2_ through ANOVA test and Bonferroni Multiple Comparison post-test (n = 10/group).

To evaluate the RNS, two molecules were quantified: NO and ONOO-. The behavior of the SIRT1 cell signaling pathway and the antioxidant effect of RSV was also evaluated (Fig. 3).

In the NO analysis shown in Fig. 3A, it is possible to observe that in the group without AD, the levels of this RNS had a significant increase when exposed to H_2_O_2_ and treated with RSV, when compared to the group exposed only to H_2_O_2_. There was also an increase when the cells were stimulated with H_2_O_2_ and the SIRT1 pathway inhibitor, when compared to the group exposed only with H_2_O_2._ There was a significant increase in NO production when the group was stimulated with the triad (sirtinol + H_2_O_2_+RSV) when compared to the H_2_O_2_+RSV group. That is, the SIRT1 pathway is important for RSV to decrease this RNS in leukocytes from elderly people without AD.

Fig. 3B shows cells from elderly people with AD. It is only possible to observe a significant increase of NO in the group exposed with H_2_O_2_ and sirtinol when compared to the group exposed only with H_2_O_2_. In addition, when comparing the studied groups, it is noticed that the NO concentration of NO in leukocytes from AD donors has higher baseline values in relation to the group without the disease. The group without stimulation has higher NO production in the presence of the sirtinol and H_2_O_2_ inhibitor, when compared to the group without the disease. Even in the presence of sirtinol, NO production is elevated in AD, mainly in the presence of the inhibitor together with H_2_O_2_.

Fig. 3C and D shows the analysis of the second RNS. In Fig. 3C, it is possible to observe that there was an increase in ONOO-stimulated with H_2_O_2_ compared to the group without any stimulus. A reduction of ONOO- is also observed when exposed with H_2_O_2_ and treated with RSV, when compared to the group exposed only with H_2_O_2_. The same reduction was also noticed when the cells were stimulated with H_2_O_2_ and the SIRT1 pathway inhibitor compared to the group with only H_2_O_2_.

In Fig. 3D, there was no significant difference between the AD leukocyte groups. However, when comparing the two groups, we observed that in donors with Alzheimer's the production of ONOO- was lower than in the group without the disease, mainly in the group without any stimulus and stimulated with H_2_O_2_.

### 3.4 Patients with AD show a correlation between increased oxidative stress and loss of functionality

In order to assess the functional profile of the elderly in the absence and presence of Alzheimer's disease, we used the Katz Index instrument, whose purpose is to classify the individual in terms of dependence (moderate or very dependent) or independence. It was possible to observe that in the group of elderly people without AD the classification was between 5 and 6, which corresponds to Independence. In the AD group, we observed a dependence profile: 1 elderly person was classified as “moderately dependent” (Katz = 4) and the other 7 elderly people were classified as “very dependent” (Katz between 0 and 1).

Finally, to analyze the influence of AD on functional capacity and on biochemical parameters of oxidative stress, we compared data from the Katz index and baseline values of leukocytes from donors. Analyzing the values of the Katz Index (Table 1), there was a significant reduction in functionality in the elderly with AD, when compared with the elderly without AD. As for the parameters of oxidative stress (ROS, NO and ONOO-), we noticed that in the leukocytes of the DA donors there was a significant increase in the baseline values of ROS and NO, while there was a reduction in the values of ONOO-, when compared with the leukocytes of the group of donors without the disease. With regard to catalase and GPx enzymes, a significant reduction in leukocytes in elderly AD patients was observed when compared to elderly individuals without the disease.

**Table 1:** Comparative analysis of functional capacity and oxidative parameters in leukocytes of elderly people with and without AD.

Parameter	Mean ± SD		
	Without Alzheimer	Alzheimer	p value
**Katz Index**	6 ± 0	0.875 ± 0.479	∗∗∗∗<0.001
**Reactive Oxigen Species**	437.00 ± 56	1137 ± 114	∗∗0.0012
**Nitric Oxide**	0.032 ± 0.001	0.189 ± 0.01	∗∗∗∗<0.001
**Peroxynitrite**	0.668 ± 0.01	0.456 ± 0.01	∗∗∗∗<0.001
**Catalase**	28.18 ± 1.83	16.98 ± 3.72	∗0.0291
**Glutathione Peroxidase**	144.7 ± 8.85	91.51 ± 8.89	∗∗0.0023

Where ∗p < 0.05; ∗∗p < 0.01; ∗∗∗p < 0.001 and ∗∗∗∗p < 0.0001 considered significant through the ANOVA test and Mann-Whitney post-test (n = 10/group).

## 4 Discussion

One important metabolic point of Alzheimer's and the focus of this study is the generation of oxidative stress (ROS and RNS), caused by the imbalance between oxidant and antioxidant species. Thus, the theory of free radicals observed both in aging and in its correlated pathologies mentions that the structural damage that predicts functional losses associated with aging is due to the accumulation of oxidative damage to macromolecules: lipids, DNA, and proteins [25]. Thus, the need arises to relate oxidative stress to AD. The interest in deepening the study of several antioxidant compounds has increased significantly in recent years. In this scenario, resveratrol has been highlighted in several studies that seek to analyze its biological effects, especially the antioxidant, anti-inflammatory, anti-aging and antitumor effects [15].

An increase in oxidative load has been reported in brains of older adults with AD [26]. Currently, many blood markers of oxidative stress have been identified in AD patients or animal models. However, it is not yet known where oxidative stress originates in this disease. Research has suggested that mitochondrial dysfunction, metal accumulation, tau protein hyperphosphorylation, inflammation and β-amyloid (βA) accumulation are the basic mechanisms underlying the induction of oxidative stress [27,28]. Aerobic organisms have integrated antioxidant systems, which include enzymatic and non-enzymatic antioxidants that are generally effective in blocking the deleterious effects of ROS/RNS. Superoxide dismutase (SOD), Glutathione peroxidase (GPx) and catalase (Fig. 1) are the key antioxidant enzymes of this defense system by which reactive species produced during metabolic reactions are removed [29].

In a review, Schrag et al. [30] analyzed markers of oxidative stress and antioxidants in the blood of individuals with AD or mild cognitive impairment. The authors observed an increase in markers of lipid peroxidation in the blood of patients with AD and mild cognitive impairment. Furthermore, copper metabolism is dysregulated, and total antioxidant capacity is decreased. Although none of the main antioxidant enzymes are decreased, blood levels of non-enzymatic antioxidants (particularly uric acid, vitamins A, E, and C, and α- and β-carotene) are significantly reduced. In summary, the authors observed in this meta-analysis that there is significant oxidative damage in peripheral blood early in the neurodegeneration process. Our findings corroborate these authors and additionally show that resveratrol has the ability to reduce ROS and increase, at least, the antioxidant action of glutathione peroxidase.

Sirivarasai et al. [31] revealed that some existing polymorphisms in the gene that encodes catalase in erythrocytes may be responsible for changing the expression and activity of this enzyme in blood pressure in a Thai population. Emamgholipour et al. [32] demonstrated that mRNA expression and catalase activity increased in mononuclease cells exposed to H_2_O_2_ compared to untreated cells. One of the current hypotheses about the mechanism of oxidative damage stimulated by amyloid β in cells in Alzheimer's patients is that it interacts directly with catalase, binding to the protein and deactivating its catalytic activity, creating an oxidative environment. Therefore, catalase has a direct relationship with the pathogenesis of Alzheimer's disease [33].

RSV is both a ROS scavenger and a potent antioxidant due to its ability to promote the activity of many antioxidant enzymes. Concomitant with the results found in the literature relating the activity of catalysis and GPx with oxidative stress, there are also studies relating the activity of these enzymes to chronic degenerative diseases in aging. Srivastava [34] shows the role of free radicals in aging, demonstrating that the oxidative stress framework increases with chronological age along with the reduction in the levels and activity of these antioxidant enzymes. Truong et al. [35] demonstrated that RSV treatment increased the expression of catalase and GPx in aortic segments of rats and the hepatic antioxidant capacity via catalase, GPx and SOD. In this study, the results of antioxidant enzymes are similar to some results found in the literature but contradict many others. According to Emamgholipour et al. [32] cells respond to the oxidative challenge with selective induction of antioxidant enzymes, stating that there is no consensus in the literature on the pattern of antioxidant enzymes in response to oxidative stress. This is probably related to the type of cell model used, the type and concentration of the oxidizing agent used, in addition to the treatment time.

Evaluating the production of ROS (Fig. 2) we observed that RSV had an antioxidant effect both in leukocytes from donors without and with AD. However, RSV acted more significantly in the group without Alzheimer's. This effect is possibly explained due to the oxidative stress present not only in aging, but associated with AD. Such results agree with the studies by Lara et al. [36], who verified the action of RSV on the Neuro-2A cell line, which had a reduction in ROS production when stimulated with RSV. Lin et al. [37] also showed that pre-treatment of astrocytes with RSV was able to suppress ROS generation, in addition to reducing the rate of cell death, which had been induced by glutamate. Bobermin et al. [38] observed a decrease in ammonia-induced ROS production from SH-SY5Y cells (human neuroblastoma cell line) pre-incubated with RSV. Caldeira et al. [39] also confirmed the preventive profile of this polyphenol, when they found the antioxidant effect of RSV on mononuclear cells in the middle-aged group compared to the elderly group.

In addition to our study, the cell signaling pathway chosen was SIRT1 (Fig. 2). The choice was made because the involvement of RSV in this way is reported in the literature. RSV is an activator of SIRT1 [39]. SIRT1 deacetylates histone and non-histone proteins, including transcription factors. In this case, pathways regulated by SIRT1 affect metabolism, resistance to oxidative stress, cell survival and senescence, mitochondrial biogenesis and genomic stability [15,40]. Souza et al. [3] observed that RSV was able to protect neuroblastoma cells against oxidative stress, increasing cell viability. However, when SIRT1 was inhibited with sirtinol administration, the antioxidant activity of RSV was suppressed. In PC12 cells, RSV increased cell viability, reduced apoptosis, and attenuated βA-induced neurotoxicity.

A key cell signalling pathway that resveratrol appears to play in aging and related diseases is the Nrf2 (nuclear factor erythroid 2-related factor 2) pathway. Nrf2 is a protein that acts as a transcription factor, i.e., it controls the expression of other genes. It is the main regulator of the body's antioxidant response, protecting cells against oxidative stress, damage, and inflammation. Studies show that this pathway is activated by RSV in the elderly and is one of the few responsible for reducing ROS and inflammatory markers [41,42].

As previously mentioned, AD is characterized by a complex interplay of pathological processes involving oxidative stress, impaired signaling pathways, and protein aggregation. Recent research has focused on identifying specific NOX isoforms involved in AD and exploring the potential of isoform-targeted therapies [43,44]. Simultaneously, the phosphatidylinositol 3-kinase (PI3K)/protein kinase B (Akt) signaling pathway, critical for neuronal survival, is consistently downregulated in AD. This decreased activity makes neurons more vulnerable to Aβ toxicity and tau hyperphosphorylation, impairing survival mechanisms and exacerbating apoptosis and synaptic deterioration. Astragalin, a natural compound, has demonstrated neuroprotective effects in APP/PS1 mice by activating the PI3K/Akt pathway and enhancing mTOR-mediated autophagy. The latter is a key cellular clearance process [45]. Furthermore, protein kinase C (PKC) plays a multifaceted role in AD, particularly in Aβ protein processing. Modulation of PKC activity by phytochemicals has emerged as a promising therapeutic avenue, as proper regulation of PKC can influence Aβ protein processing and reduce its accumulation [46].

Disruptions in the PI3K/Akt signaling pathway, a key regulator of neuronal survival, growth, and metabolism, are implicated in the pathogenesis of AD. Phytochemicals, including resveratrol, curcumin, berberine, and quercetin, demonstrate the ability to modulate this pathway and offer potential therapeutic benefits. Curcumin promotes neuronal survival and reduces oxidative stress by directly activating PI3K/Akt. Resveratrol, by increasing SIRT1 activity, indirectly activates PI3K/Akt, providing neuroprotection. Berberine protects neurons against oxidative stress, inflammation, and apoptosis through the combined activation of PI3K/Akt and Nrf2. Furthermore, quercetin's modulation of PKC signalling pathways suggests a role in mitigating neurodegenerative processes [47,48].

In Fig. 3, we observe the role of RSV in modulating NO levels only in the group without AD. Furthermore, NO levels are higher at baseline in the group with the presence of neuroinflammation. Our hypothesis is that this reactive species also behaves as a neuromodulator and that it is at its limit of action. It is worth remembering that NO is a short-lived gaseous free radical secreted by the endothelium and that acts as a pleiotropic intracellular messenger, exerting a variety of biological actions in physiological and pathological conditions. While in low concentrations they are beneficial for several physiological and cellular functions, such as maintaining muscle tone, coagulation and inflammation well balanced and controlled; however, inits high concentrations can cause harmful and deleterious effects [49].

According to Machado et al. [50] ROS and RNS play a key role in neuroinflammation, found in Alzheimer's. However, Venturelli et al. [51] showed that in humans the depletion of NO availability is correlated with the reduction of cortical, extracranial and peripheral blood flow during aging and in parallel with the severity of Alzheimer's disease. This study corroborates our results.

In the AD group, there was no statistical difference between the treated groups. However, comparing AD and the group without AD, NO concentrations were higher. NO production may be elevated due to neuroinflammation. Souza et al. [3] showed that NO overproduction was associated with Alzheimer's Disease, resulting in damage to DNA and mitochondrial structure. Agostinho et al. [52] mentions that oxidative stress and/or other toxic substances can trigger the overexpression of nitric oxide synthase (iNOS) leading to an increase of NO in the central nervous system.

RSV could not decrease NO production (which was high) in the group with Alzheimer's, possibly due to apoptosis, a situation in which polyphenol is no longer able to exert its antioxidant function. Machado et al. [50] observed an accumulation of beta amyloid plaque as one of those responsible for chronic inflammation and activation of the immune system. Similar studies corroborate this result, such as the work by Correia-Costa et al. [53], who showed an increase in oxidant status (leading to increased cardiometabolic risk) and NO (as a negative impact on kidney function) in overweight and obese children.

The increase in NO in neurodegenerative diseases can be explained by the production of peroxynitrite (ONOO^−^) due to a direct reaction with NO. According to Pacher et al. [54], many deleterious toxic effects found in AD may be mediated by its oxidized products, rather than by NO itself. More recent evidence indicates that most of the cytotoxicity attributed to NO comes from ONOO- produced by the controlled diffusion reaction between NO and another free radical, O_2_^•-^. Peroxynitrite can interact directly with lipids, DNA and proteins via direct oxidative reactions or via indirect mechanisms mediated by other radicals. Such reactions can trigger cellular responses, ranging from subtle modulations in cell signaling to intense oxidative damage, leading cells to death by necrosis or apoptosis.

Our hypothesis is that one of the consequences of the oxidative stress present in AD leads to a functional decline. Ferreira et al. [24] describes that the functional assessment has been essential for assessing the health of the elderly. Thus, we sought to analyze the influence of functional capacity in groups of elderly people without and with Alzheimer's (Table 1). In the AD group, there was a significant reduction in functionality, observed through the low values of the Katz Index, when compared to the group without Alzheimer's. We observed that the greater the cognitive impairment observed in Alzheimer's, the lower its functional capacity, according to the instrument chosen for this study. Our data agree with the findings of Ferreira et al. [24] demonstrating that elderly people with Alzheimer's were more dependent when compared to elderly people without the disease, using the same functional instrument. With the intention of comparing the functionality with the parameters of oxidative stress, we observed a reduction in the functional capacity in the elderly with Alzheimer's disease concomitant to the decrease of the antioxidant defences, leading to a picture of oxidative stress.

For this, literature shows that resveratrol has been increasingly used as an adjuvant therapy in some diseases, whether for prevention or treatment [[9], [10], [11], [12], [13], [14], [15], [16]]. Research on natural compounds points to resveratrol as one of the most promising compounds in AD research, along with curcumin, berberine and quercetin [47,48]. Its biological effects include cardioprotective, anticancer, antiobesity, neuroprotective (Alzheimer's and Parkinson's), anti-inflammatory, antidiabetic and antioxidant effects [[9], [10], [11], [12], [13], [14]]. It is worth noting that further studies on the role of this polyphenol are still needed, such as trials involving the inhibition of other cell signalling pathways. A previous study by our group demonstrated, for example, that Alzheimer's inhibits the AMPK, resulting in a significant loss of RSV's antioxidant effect [17]. Other pathways need to be analyzed, as well as their direct relationship with the formation of Aβ plaques, hyperphosphorylation of tau protein and, consequently, the loss of synapses and neurons.

## 5 Conclusion

In this study, we found that RSV had an antioxidant effect on the leukocytes of both donors, however its action was less effective in donors with Alzheimer's, despite the SIRT1 cell signaling pathway having been shown to be active in the disease. More studies are needed to understand which other antioxidant pathways may be inhibited in AD, contributing to oxidative stress and lower antioxidant effect of RSV. In addition, elderly people with the disease are less functional. Therefore, taken together, our study corroborates the hypothesis that oxidative stress present in the disease predisposes to increased loss of autonomy and dependence. With this, it is expected that these results can contribute to studies related to the treatment of Alzheimer's disease, both at the biochemical and functional level.

## CRediT authorship contribution statement

**Filipe Nogueira Franco:** Writing – review & editing, Writing – original draft, Formal analysis, Conceptualization. **Luciana de Cassia Cardoso:** Methodology, Investigation, Formal analysis, Data curation, Conceptualization. **Bárbara Néllita Moura Silva:** Methodology, Investigation, Formal analysis, Data curation, Conceptualization. **Glaucy Rodrigues de Araújo:** Writing – review & editing, Supervision, Project administration. **Miriam Martins Chaves:** Writing – review & editing, Funding acquisition.

## Funding

This research was supported by the Fundação de Amparo Pesquisa do Estado de Minas Gerais (10.13039/501100004901FAPEMIG – APQ-02574-14), Conselho Nacional de Desenvolvimento Científico e Tecnológico (10.13039/501100003593CNPq), Coordenação de Aperfeiçoamento de Pessoal de Nível superior (10.13039/501100002322CAPES) and 10.13039/501100007374Universidade Federal de Minas Gerais (PRPq/10.13039/501100007374UFMG).

## Declaration of competing interest

The authors declare that there was no conflict of interest.

## Data Availability

Data will be made available on request.
